# Antepartum Diagnosis and Management of Lamin A/C Disease

**DOI:** 10.1155/2019/3512706

**Published:** 2019-11-11

**Authors:** Nosheen Reza, Jessica L. Chowns, Amy Marzolf, Jessica Kim, Lisa D. Levine, Gregory Supple, Anjali Tiku Owens

**Affiliations:** ^1^Center for Inherited Cardiovascular Disease, Division of Cardiovascular Medicine, Perelman School of Medicine, University of Pennsylvania, Philadelphia, PA, USA; ^2^Maternal and Child Health Research Center, Department of Obstetrics and Gynecology, Perelman School of Medicine, University of Pennsylvania, Philadelphia, PA, USA; ^3^Cardiac Electrophysiology Section, Division of Cardiovascular Medicine, Perelman School of Medicine, University of Pennsylvania, Philadelphia, PA, USA

## Abstract

Lamin A/C cardiac disease is a genetic cardiomyopathy and arrhythmia syndrome caused by alterations in the function of the nuclear lamin A and C proteins. It is inherited in an autosomal dominant manner and usually presents in mid- to late adulthood with atrioventricular conduction abnormalities, atrial and ventricular arrhythmias, biventricular dysfunction, and advanced heart failure. While rare, women of childbearing age can exhibit an aggressive disease course, and appropriate risk stratification and management are critical. Here, we present a case of newly diagnosed lamin A/C cardiac disease in a pregnant woman.

## 1. Introduction

The majority of arrhythmias in pregnancy are benign, but the recognition of arrhythmias with high-risk features is critical to the avoidance of adverse maternal and fetal outcomes. We report this rare case of the antepartum diagnosis and management of lamin A/C disease.

## 2. Case Report

A 33-year-old woman presented to our electrophysiology clinic for evaluation of 1^st^ degree atrioventricular (AV) delay. She had been diagnosed with this finding 2 years prior while undergoing biannual fitness testing in the Navy. At that time, she had undergone Holter monitoring and treadmill stress echocardiography that did not show malignant conduction disease, arrhythmias, or structural heart disease.

**Figure 1:**
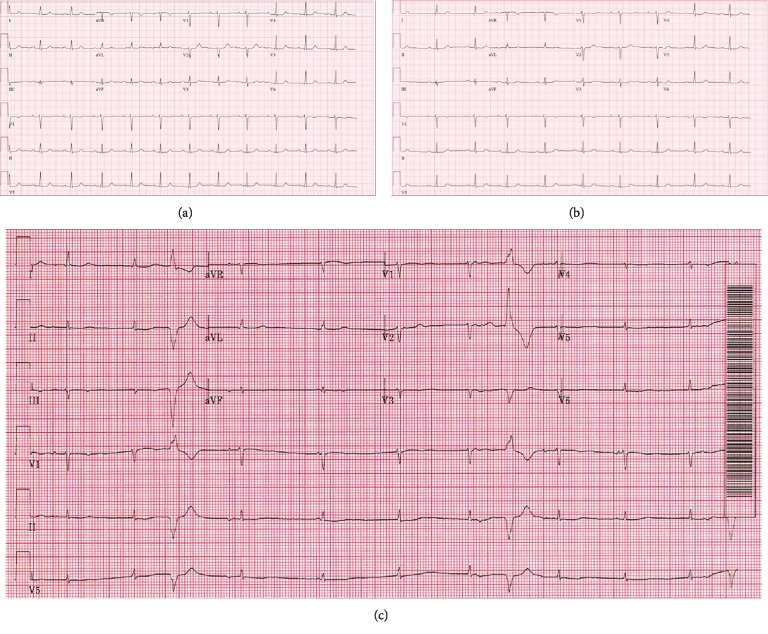
Serial ECGs: initial ECG performed at age 33 (a) with sinus rhythm and prolonged PR interval. Surveillance ECG performed one year later at age 34 (b) with slowing of the sinus rate and prolonged PR interval. First ECG during pregnancy performed at age 35 (c) showed new sinus bradycardia with competing junctional rhythm, occasional PVCs (RBS axis), and lower voltage QRS and poor R wave progression. Paper speed and amplification: 25 mm/s and 1 mV/10 mm.

At this electrophysiology clinic visit, she reported mild exercise intolerance and a sense that her “heart was not keeping up,” which she attributed to physical deconditioning. Her electrocardiogram (ECG) was notable for sinus rhythm at 71 beats per minute with a PR interval of 214 milliseconds and was otherwise unremarkable (Figure 1(a)). She underwent repeat treadmill stress testing to assess her heart rate response and AV conduction; she achieved 78% of the maximum predicted heart rate and had superiorly directed premature ventricular contractions (PVCs) and slow accelerated idioventricular rhythm during recovery. Cardiac magnetic resonance imaging, performed to evaluate for infiltrative disease, showed normal biventricular function, normal chamber structure and dimensions, and no late gadolinium enhancement. She was diagnosed with high vagal tone.

**Figure 2:**
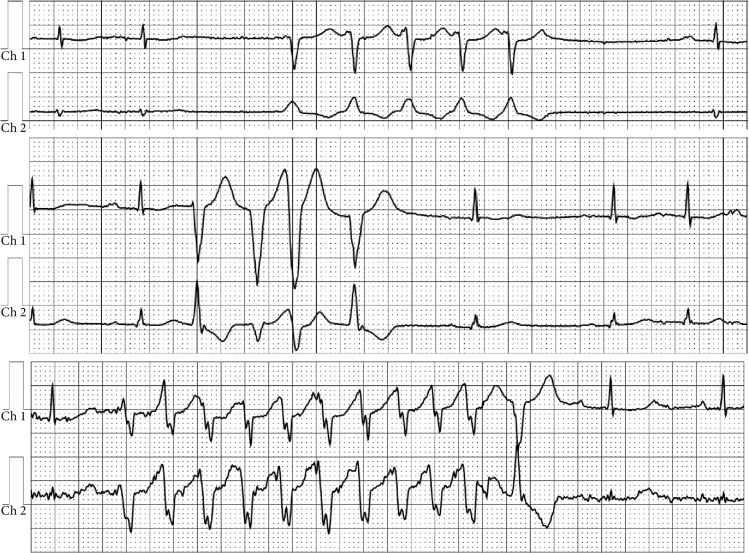
Mobile cardiac outpatient telemetry: frequent multifocal ventricular arrhythmias.

At her next follow-up two years later at age 35, she was 22 weeks pregnant with her first child. She reported becoming easily dyspneic with exercise in the context of a 10-15-pound weight gain and intermittent palpitations. Her ECG showed sinus bradycardia, a competing junctional rhythm, occasional PVCs, and poor R wave progression—all of which were new (Figure 1(c)). Review of her family history revealed that her father died at age 47 while awaiting heart transplantation for presumed end-stage ischemic cardiomyopathy. Given these findings, a 30-day ambulatory arrhythmia monitor was ordered, and within the first two weeks, sinus node dysfunction and frequent multifocal ventricular arrhythmias were captured (Figure 2). In the setting of both her tachy- and bradyarrhythmias and concerning family history, a genetic disorder was suspected. She underwent urgent and successful dual-chamber implantable cardioverter defibrillator (ICD) implantation at 25 weeks gestation, was started on metoprolol, and established care with our Maternal-Fetal Medicine program.

**Figure 3:**
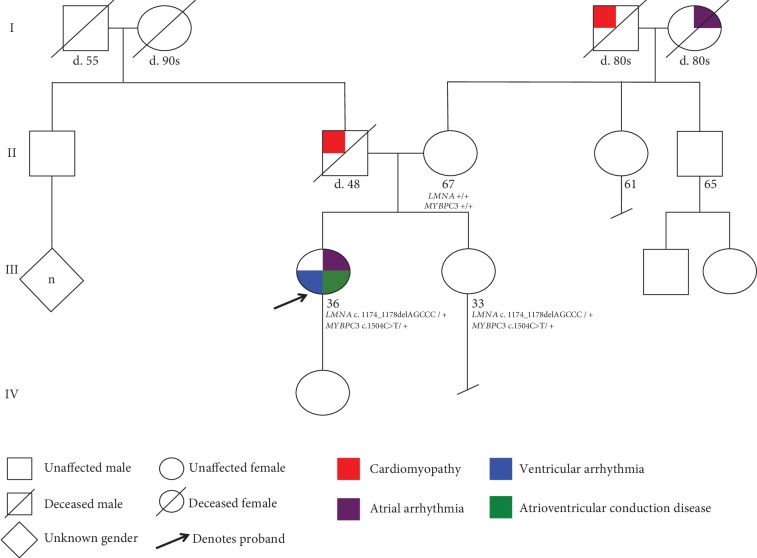
Pedigree: numbers below symbols denote current age or age at death. + denotes normal allele.

She was referred to our inherited cardiovascular disease center for genetic counseling and testing. Her three-generation family history was notable for cardiomyopathy in her father and heart issues in her paternal grandfather (Figure 3). She underwent genetic sequencing of 121 genes associated with cardiomyopathy and arrhythmia through GeneDx (Gaithersburg, Maryland) and was found to be heterozygous for two different pathogenic variants—a frameshift variant in *LMNA* (c.1174_1178delAGCCC; p.Ser392TyrfsX32) and a missense variant in *MYBPC3* (c.1504C>T; p.Arg502Trp). This specific *LMNA* variant had not previously been reported but was predicted to result in either protein truncation or loss of protein product through nonsense-mediated mRNA decay and was therefore felt to be the likely cause of her arrhythmias. The *MYBPC3* (c.1504C>T) variant had previously been reported in multiple individuals in association with hypertrophic cardiomyopathy. Cascade screening of first-degree relatives was recommended.

At 32 weeks gestation, device interrogation and manual review of reported events showed several prolonged episodes of atrial fibrillation. After extensive discussion regarding the risks and benefits of therapeutic anticoagulation, she was started on prophylactic enoxaparin with the plan to undergo induction of labor at term (37 weeks gestation). She underwent an uncomplicated low transverse cesarean section due to nonreassuring fetal heart tones during her induction. She was discharged on therapeutic enoxaparin for thromboprophylaxis given limited data on the use of direct oral anticoagulants during breastfeeding.

## 3. Discussion

The lamin A/C gene (*LMNA*) is located on chromosome 1q21-22 and encodes two main nuclear laminar proteins, lamin A and lamin C, by alternative splicing [1]. These type V intermediate filament proteins are expressed in terminally differentiated somatic cells and are required for normal formation of the cell's nuclear envelope. When these proteins are disrupted, the stability of the nuclear membrane along with DNA replication, transcription, and nuclear organization are threatened [2]. Lamin A/C cardiac disease is inherited in an autosomal dominant pattern; therefore, heterozygotes often manifest disease, although with variable expressivity.

Diseases caused by variants in lamin A/C are collectively termed laminopathies and affect skeletal muscle (e.g., autosomal dominant and recessive Emery-Dreifuss muscular dystrophy, autosomal dominant limb-girdle muscular dystrophy), adipose tissue (e.g., familial partial lipodystrophy), peripheral nervous tissue (e.g., sensory and motor axonal neuropathy), and cardiac muscle [1]. There is wide variability among these phenotypes, with some patients first and only displaying cardiac manifestations characterized by AV conduction abnormalities, arrhythmias, biventricular dysfunction, and advanced heart failure. In addition, the majority of pathogenic *LMNA* variant carriers with muscular dystrophy-predominant symptoms also develop cardiac complications. Natural history studies have shown that conduction disease without other structural heart disease develops in a majority of these patients by the age of 30 with heart failure mostly manifesting around 20 years later [3]. Variants in *LMNA* have been found in 5-8% of familial dilated cardiomyopathy (DCM) cases, making these variants among the most frequently implicated in the development of DCM [1, 2, 4]. Our patient did not exhibit any other laminopathic signs or symptoms, including neuromuscular symptoms, and had stable left ventricular structure and function on her serial third trimester and postpartum transthoracic echocardiograms. She will remain under periodic echocardiographic surveillance.

Conduction disturbances associated with *LMNA* include atrial and ventricular arrhythmias and AV conduction delays and blocks. Typically, sinoatrial disease becomes apparent first and manifests as sinus bradycardia, sick sinus syndrome with sinus arrest and junctional escape rhythms, and first-degree AV delay. Supraventricular arrhythmias include atrial fibrillation, atrial flutter, and atrial tachycardia. In a multicenter international study of *LMNA* pathogenic or likely pathogenic variant carriers, 45% of patients who had atrial fibrillation on presentation progressed to persistent or permanent forms within the median follow-up period of 7 years [5]. In comparison to genotype-negative idiopathic DCM patients, *LMNA* variant carriers experienced a significantly higher risk of arterial and venous thromboembolic complications (HR 4.8, 95% CI: 2.2-10.6), raising the possibility of an additional prothrombotic phenotype [6]. Pregnancy is, in itself, a hypercoagulable state, and this combined with our patient's episodes of atrial fibrillation necessitated the consideration of systemic anticoagulation and delivery.

*LMNA* carriers have significantly worse cardiovascular outcomes compared to other DCM patients with increased mortality due to sudden cardiac death and severe heart failure [1]. In a multicenter European study of 269 *LMNA* variant carriers, independent risk factors for malignant ventricular arrhythmias included nonsustained ventricular tachycardia (VT), left ventricular ejection fraction < 45% at first clinical contact, male sex, and nonmissense (i.e., insertion, deletion, truncations, and splice site) variants [7]. In this same study, there was a high rate of appropriate ICD therapies in patients who had received a primary prevention device (8 per 100 person-years with a median follow-up period of 29 months). Similarly, in another study, 42% of patients with genetically confirmed lamin A/C disease and primary prevention ICDs sustained appropriate shocks [8]. These data are the foundation for the 2015 European Society of Cardiology guideline class IIa recommendation to consider ICD implantation in patients with DCM, a confirmed disease-causing *LMNA* variant, and clinical risk factors [9].

Our patient's known pathogenic variant, positive family history, and documented progressive conduction disease with new and frequent multifocal ventricular arrhythmias were thought to confer a high enough risk of sudden cardiac death to warrant primary prevention ICD implantation, regardless of her pregnant state. The risks of bradycardia and asystole were also important considerations in implanting a pacing system given that these rhythms could be acutely life threatening to the patient and fetus. As the patient was already at 25 weeks gestation at the time of device implantation, the risks of radiation exposure to the fetus were somewhat attenuated. A dual-chamber device can be implanted with very low dose fluoroscopy, as was the case here, with only 2 minutes of low frame rate fluoroscopy used. A significantly higher fluoroscopy dose would have been used to implant a cardiac resynchronization therapy lead; therefore, her device was implanted with appropriate programming to minimize right ventricular pacing.

Data on catheter ablation for management of ventricular arrhythmias in lamin A/C disease are limited. In a recent international multicenter study of 25 *LMNA* patients with drug-refractory VT, catheter ablation was associated with poor procedural success and a very high rate of ventricular arrhythmia recurrence. Notably, at a follow-up, 44% of patients were on or awaiting advanced therapies for end-stage heart failure, and 26% died [10]. Catheter ablation is mostly palliative, and further prospective studies are needed to assess whether guideline-directed medical therapy can delay the onset and progression of advanced heart failure. At this time, heart transplantation is the only curative therapy for lamin A/C cardiac disease. A phase 3, randomized, placebo-controlled clinical trial evaluating the safety and efficacy of a selective oral inhibitor of p32 mitogen-activated protein kinase in treating patients with symptomatic *LMNA* DCM (NCT03439514) is currently enrolling and expected to reach study completion in July 2020 [11].

Arrhythmias are one of the most common cardiac conditions in pregnancy. Contributors to this proarrhythmic state include myocardial stretch due to a 50% increase in blood volume, hormonal changes, and an increase in circulating catecholamines [12]. Supraventricular tachycardias are thought to be the most frequent sustained arrhythmias that women experience during this time [13]. Sustained VT and ventricular fibrillation are quite rare in pregnancy with registry data showing a 1.4% incidence in women with underlying structural heart disease [14].

Data on obstetrical and fetal outcomes in lamin A/C cardiac disease are exceedingly scarce. The only published case series describes 11 pregnancies in 5 *LMNA* variant carriers. Of the 5 women, 4 had palpitations or AV conduction abnormalities prior to or during their pregnancies. No major cardiac events were observed during their pregnancies. Unfortunately, no systematic electrocardiographic data were reported, precluding direct comparison with our patient [15].

The proband and her sister (Figure 3, individual III-3) were both heterozygous for disease-associated variants in *LMNA* and *MYBPC3*. Although the clinical lab interpreted the *MYBPC3* c.1504C>T variant as pathogenic, the variant has conflicting interpretations of pathogenicity in ClinVar [16]. At this time, the proband is only exhibiting a phenotype consistent with the pathogenic *LMNA* variant; her left ventricular wall thickness is normal. Individuals with *LMNA*-related cardiac disease generally exhibit a highly penetrant phenotype, consistent with the proband's presentation and course, while penetrance is reduced for *MYBPC3* variants. The proband and her sister will remain under clinical surveillance for manifestations of *LMNA*-related cardiac disease and of hypertrophic cardiomyopathy. At this time, the proband's sister's clinical phenotyping, consisting of a physical exam, electrocardiogram, and echocardiogram performed at another institution, shows no evidence of disease. She is scheduled to undergo additional phenotyping with cardiac magnetic resonance imaging and ambulatory electrocardiographic monitoring.

To our knowledge, this is the first report of the diagnosis and management of lamin A/C cardiac disease progression during pregnancy. Our patient experienced a typical disease course as described above with progressive sinoatrial and AV abnormalities and ultimately atrial and ventricular tachyarrhythmias necessitating dedicated management, but whether this course was hastened by her pregnancy remains unclear.

## 4. Conclusions

Progressive conduction system disease in a young individual is a hallmark of lamin A/C disease. With the mean age of mothers at first birth in the United States rising, it is likely that we will see genetic cardiomyopathies like lamin A/C disease, thought usually to present in mid- to late adulthood, become manifest in antepartum and postpartum women [17]. Cardiologists should be aware of *LMNA*-related cardiac disease and the importance of electrocardiographic monitoring in the diagnosis of inherited arrhythmia and cardiomyopathy syndromes.
